# Nano-Based Approaches in Surface Modifications of Dental Implants: A Literature Review

**DOI:** 10.3390/molecules29133061

**Published:** 2024-06-27

**Authors:** Chrysa Marasli, Hector Katifelis, Maria Gazouli, Nefeli Lagopati

**Affiliations:** 1 Laboratory of Biology, Department of Basic Medical Sciences, Medical School, National and Kapodistrian University of Athens, 11527 Athens, Greecemgazouli@med.uoa.gr (M.G.); 2 School of Science and Technology, Hellenic Open University, 26335 Patra, Greece; 3 Biomedical Research Foundation, Academy of Athens, 11527 Athens, Greece

**Keywords:** nanomaterials, implantology, osseointegration, surface modifications, biomimicry, immunomodulation, peri-implantitis

## Abstract

Rehabilitation of fully or partially edentulous patients with dental implants represents one of the most frequently used surgical procedures. The work of Branemark, who observed that a piece of titanium embedded in rabbit bone became firmly attached and difficult to remove, introduced the concept of osseointegration and revolutionized modern dentistry. Since then, an ever-growing need for improved implant materials towards enhanced material–tissue integration has emerged. There is a strong belief that nanoscale materials will produce a superior generation of implants with high efficiency, low cost, and high volume. The aim of this review is to explore the contribution of nanomaterials in implantology. A variety of nanomaterials have been proposed as potential candidates for implant surface customization. They can have inherent antibacterial properties, provide enhanced conditions for osseointegration, or act as reservoirs for biomolecules and drugs. Titania nanotubes alone or in combination with biological agents or drugs are used for enhanced tissue integration in dental implants. Regarding immunomodulation and in order to avoid implant rejection, titania nanotubes, graphene, and biopolymers have successfully been utilized, sometimes loaded with anti-inflammatory agents and extracellular vesicles. Peri-implantitis prevention can be achieved through the inherent antibacterial properties of metal nanoparticles and chitosan or hybrid coatings bearing antibiotic substances. For improved corrosion resistance various materials have been explored. However, even though these modifications have shown promising results, future research is necessary to assess their clinical behavior in humans and proceed to widespread commercialization.

## 1. Introduction

Dental implants aim to optimally restore masticatory function and improve the quality of life in partially or fully edentulous patients by offering stability to the overlying prosthetic restoration [1]. Their application is mainly based on the concept of osseointegration, the connection between living bone and material, as described by Branemark in his experiments in the mid-1960s [2]. The interface between tissue and implant material is pivotal for the determination of the final clinical outcome and the success of the treatment. Chemical, physical, mechanical, and topographic characteristics of the implant surface influence the healing process taking place after insertion into the bone [3,4]. Researchers aim to improve the tissue–material fusion by altering the characteristics of the implant material and surface in order to achieve faster and superior osseointegration. The first experimentation conducted was the macroscopic modification of the implant design. The length, diameter, morphology, and thread design influence on the success of the implant were analyzed and thoroughly studied [5]. Micromodifications and surface coatings were later applied in order to have an impact on the connection at the cellular level, as osteoblast differentiation is shown to be dependent on surface characteristics and roughness [6].

Modern technologies shifted the research from macro to nano surface modifications. Nanotechnology opened a new era for the rapidly growing implant market by introducing new nanostructured materials and coatings, as well as manufacturing techniques. Nanostructured surfaces are shown to have an impact on both indirect and direct cell interactions, guiding specific molecular events [7]. For the indirect mechanism of protein adsorption, a study by Webster and his team demonstrated increased vitronectin adsorption in nano-modified surfaces, as well as increased osteoblast adhesion when compared to fibroblast adhesion. The same study has shown increased osteoblast adhesion on nano-modified surfaces, with the affinity ratio between osteoblasts and fibroblasts being 3 to 1, while in conventional materials it is 1 to 1 [8]. This is a significant finding, as it was proved that nanostructured surfaces display selectivity in cell adhesion. The direct mechanism was also impacted as cell adhesion and motility were found altered for nano-treated surfaces [7]. On the other hand, bacteria adhesion and colonization were decreased on nanophased materials. Colon et al., investigating ZnO and titania (TiO_2_) nanostructured orthopedic implants, discovered reduced Staphylococcus epidermis adhesion levels [9]. This information bears importance, as it is thought that nano modifications in dental implants can contribute to implant longevity with the prevention of pathological entities like peri-implantitis. Nanoroughness increases the surface-to-volume area, providing ample binding sites for increased cell attachment and a more stable mechanical interlocking with native bone, leading to faster and superior osseointegration [10].

All the aforementioned properties render nanomaterials invaluable tools in implantology. Lowering the failure rate of dental implants through enhancement of osseointegration and bone healing, and reduction of infections in new implants is sought after via nanomodifications on their surfaces [10,11]. Another therapeutic target of nano-engineered implants is the enhancement of healing, especially in patients with metabolic bone disorders and systemic diseases, and the decrease in healing times allowing faster and even predictable immediate loading with the prosthetic restoration [10,12]. The prevention of peri-implantitis through antimicrobial drug loading on the surface of the implant is an objective of this technology [13]. Finally, a viable goal is the improvement of mechanical stability, stress, and corrosion resistance.

A number of recent reviews have addressed the role of nanotechnology in the construction of enhanced implant surfaces. In their review, Gulati et al. explored various nanoengineering strategies with emphasis on nanofabrication methods, showcasing that anodization seems to be superior [14]. Kunrath et al. investigated a plethora of nanoengineered materials for implant and periodontal applications [15]. However, the focus of the study was biomaterials with drug delivery systems. The aim of this review is to present the contribution of nanomaterials in the enhancement of dental implant surfaces. A synopsis of the major in vitro and in vivo studies exploiting nanomaterials will be made and their properties will be investigated. The potential weaknesses and challenges will also be highlighted.

## 2. Methods

A search of peer-reviewed journals was conducted based on relevant search terms alone or in combination, including “dental implants”, “nanotechnology”, “surface”, “modifications”, “nano”, “nanomedicine”. The two main databases used were PubMed and Google Scholar. For the main body of the thesis, papers published in English from 2003 to 2024 were selected according to relevance. Preliminary screening of papers was based on the titles and abstracts of the researched papers. Additional studies were chosen manually from the references of the papers already selected.

## 3. Nanomaterials in Implantology

### 3.1. Nanotubes

Nanotubes are one-dimensional long thin cylinders, with a length of 0.5–300 mm and diameter of 10–300 nm, formed by the folding of the contextual material sheet. They are highly ordered structures exhibiting great mechanical strength and chemical and thermal stability. Carbon (CNTs) and especially titania (TNTs) nanotubes are the ones mostly explored in implantology. CNTs are cylindrical shaped folded graphene sheets, found as single or multi-walled conformations [16,17]. TNTs are empty nanoscale test tubes, opening at the top and closing at the bottom, fabricated through electrochemical anodization on pure titanium and its alloys. TNTs can be loaded with bioactive therapeutic modules such as antibiotics, anti-inflammatory drugs, growth factors, and proteins. This way they act as reservoirs regulating controlled release of the particular agent. They are scalable and cost-effective, their characteristics, morphology, and geometry can be tuned and they can be functionalized with one or multiple agents at the same time. TNTs provide biomimetic and biocompatible surfaces for controlled cellular growth, as established by in vivo studies [17,18].

### 3.2. Nanopores

Nanopores are small holes with diameters of up to 100 nm in the nanoscale. They highly resemble nanotubes except for the fact that they do not have inter-nanotube distance. When compared to TNTs, they exhibit superior mechanical properties. This type of nanomaterial is not widely researched yet [17].

### 3.3. Metal Nanoparticles

Silver nanoparticles (AgNPs) display strong antimicrobial features against various bacterial species. There are two mechanisms involved in their mode of action, one direct and one indirect. The direct mode of action involves the adherence of silver ions on the bacterial cell wall and cytoplasmic membrane, followed by oxidation and disruption of the membrane, leading to damage of the organelles and inhibition of cell growth and cell lysis at the end. The indirect method focuses on the formation of reactive oxygen species (ROS) and the prevention of DNA replication [19,20]. Bactericidal properties are shown to be size-dependent, as small AgNPs less than 10 nm exhibit higher Ag ion release [21]. AgNPs can also promote osteogenesis and soft tissue integration. However, the release of free Ag ions and ROS raises the issue of cytotoxicity. In implantology, AgNPs are usually applied as coatings for the prevention of peri-implant diseases [20].

Zinc and zinc oxide nanoparticles (Zn/ZnO NPs), like AgNPs, present antibacterial action through ion release or generation of ROS. The application of ZnO NPs has shown increased bone growth and osteoblast proliferation [19,22,23].

Copper nanoparticles (CuNPs) have also been investigated as promising nanomaterials for implant surface modifications. They are cost-effective, stable, and can be combined with a variety of polymers. They demonstrate osteogenic, angiogenic, and long-lasting antibacterial properties and are shown to enhance the bioactivity of the given system [19].

### 3.4. Silica Nanoparticles

Silica nanoparticles are inorganic nanomaterials comprising silicon dioxide and can be distinguished as nonporous and mesoporous. They present tunable size and pore volume, easily modifiable surfaces due to the presence of silanol groups (Si-OH), and excellent biocompatibility [24]. Si-OH groups are shown to contribute to bioactivity since they can react with biological fluids. Mesoporous silica nanoparticles mainly serve as vessels for drug loading. They can be combined both with other nanomaterials and drugs to promote proliferation and differentiation of osteoblasts [19]. Their porosity allows the encapsulation of several drugs or growth factors to achieve this regenerative performance. Additionally, several molecules can be customized on their surface, transforming them into responsive drug delivery systems [25]. Their highly customizable nature renders them invaluable tools in implantology.

### 3.5. Hydroxyapatite

Hydroxyapatite (HA) is a layer of calcium and phosphate atoms deposited on the implant surface as a coating via plasma spraying technique [26,27]. It highly resembles native bone tissue in its chemical composition and crystalline structure, providing great bond stability at the implant–bone interface. The main advantages of HA coatings are their biocompatibility, osteoconductivity, and good mechanical properties [28]. Studies have shown improved primary stability at implants coated with HA, aiming at reduction of treatment time [3]. However, the HA coating formed through plasma spraying constituted a thick (40–50 μm), porous, and uneven surface leading to marginal bone resorption and eventually implant failures [3,28]. In a study by Zhou et al., lower 10-year success rates for these types of implants (63.84%) were identified when compared to overall success rates (87.39%) [29]. Additionally, early HA coatings fabricated through plasma spraying demonstrated lower bonding strength at the bone–implant interface, degradation of titanium particles to the adjacent tissues, and incompatibility with antibiotics and other agents [3,19]. To improve the coating quality, the introduction of new manufacturing techniques was required and consequently nano-HA was introduced. Hydroxyapatite nanocrystals highly resemble the size and morphology of native HA crystals in bone and can enhance the implant surface reactivity. Nano-HA can be manufactured with a variety of techniques, the most prominent of which are electrodeposition and wet chemical process [22,29]. Via those techniques, researchers were able to acquire a thin homogenous layer acting as a scaffold where other biological agents such as antibiotics, growth factors, and nanomaterials like graphene oxide (GO) and chitosan can be incorporated, leading to the formation of hybrid coatings with improved interface qualities [19,29].

### 3.6. Carbon Nanoparticles

Carbon nanomaterials, such as graphene and graphene oxide, have been widely explored for the modification of dental implant surfaces due to their excellent biocompatibility. Graphene is a chemically stable hydrophobic honeycomb monolayer acquired from the physicochemical exfoliation of graphite. Its favorable physicochemical properties render this an ideal candidate as an implant coating nanomaterial, aiming to increase the bioactivity of the host tissue and the antimicrobial properties of the implant [19]. Graphene oxide (GO) is an oxidized derivative of graphene with highly hydrophilic properties due to the presence of surface reactive oxygen species offering the ability for additional biofunctional customization. This attribute has found applications, especially in tissue engineering, as it allows carbon nanomaterials to act as a scaffold for the incorporation of a variety of agents in their core [30,31]. Enhanced osteogenic activity has been reported for implants coated with GO. An in vitro study by Di Carlo et al. on titanium discs covalently functionalized with GO has shown more prominent extracellular matrix deposition, increased osteoblast activity, and faster osteogenic differentiation, with no cytotoxic response reported whatsoever [31]. However, it is important to highlight that the toxicity of carbon nanomaterials is under investigation. Cases of cytotoxicity have been reported for pure graphene, while no significant effects took place when nano-GO was used [19]. The advantages of carbon nanomaterials such as low cost, safe preparation, increased bioactivity, and the capability for drug loading and customization make this a potential material for future implant applications [19].

### 3.7. Biopolymers

**Figure 1:**
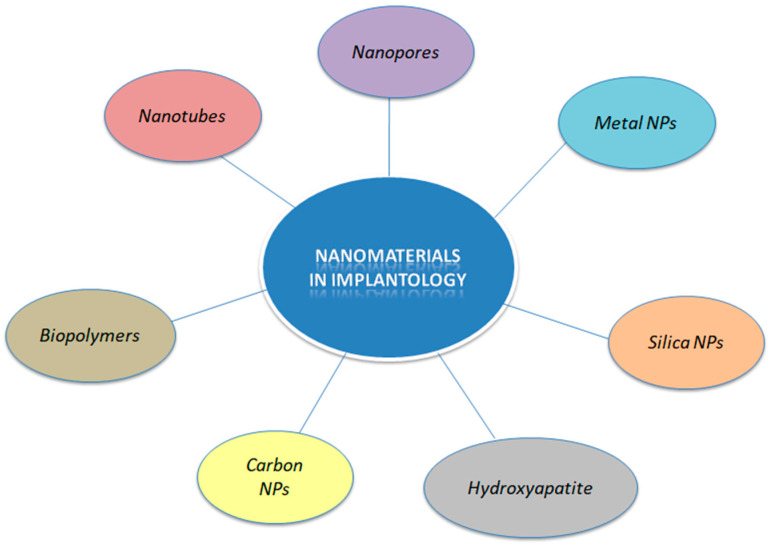
Schematic representation of nanomaterials employed in implantology.

Biopolymers have been employed as a viable strategy for dental implant modifications as they can enhance the bioactivity and act as vessels for antibacterial drug loading [19]. The most prominent polymer used in implantology is the synthetic polysaccharide chitosan stemming from the chemical N-deacetylation of chitin. Chitosan (Cht) is a biocompatible, non-toxic, and biodegradable polymer with inherent antibacterial and anti-inflammatory properties [19,32]. Nanofibrous chitosan has the unique ability of mimicking the extracellular matrix and its positive surface charge can rupture negatively charged membranes of bacteria cells, thus presenting great antibacterial function. Chitosan can be found in nanofiber and nanoparticle form, usually in combination with other nanomaterials, and can be applied in tissue engineering, controlled or sustained drug release, and immunomodulation, as it has the capacity to be loaded with particular drugs or genes in order to induce the desirable biological effect [19,32]. However, it has to be highlighted that the bond strength between chitosan and titanium is questionable, as chitosan has to be modified in order to display better adaptation on titanium surfaces. Without modification, chitosan is a cationic, hemostatic and insoluble in high pH molecule with high molecular weight. Modifications aim at the creation of water-soluble anionic molecules with preserved molecular weight and excellent viscosity. Regarding toxicity, reports are contradictory and its cytotoxic dynamic remains to be investigated. Overall, chitosan-coated dental implants present great osseointegration capacity but are under research and not commercially available yet [19,32]. Figure 1 represents the main nanomaterials that are commonly used in implantology.

## 4. Enhancement of Implant Integration

For successful implant integration, it is critical to establish sufficient bone formation along the implant surface and secure the device from the implemented masticatory forces. The implant is initially stabilized into the bone only with mechanical forces, later replaced by biological tissue formation and attachment. Soft tissue integration around the transmucosal subgingival part is crucial as it builds a barrier to external foreign attacks. Achievement of rapid and superior osseointegration is sought after in order to increase success rates and decrease the required treatment time [33]. Additionally, medically compromised patients suffering from chronic pathological conditions affecting bone metabolism and healing procedures like osteopenia, osteoporosis, and diabetes mellitus display decreased bone mass, impaired bone mineralization, and reduced bone turnover. These conditions subsequently affect implant stability and longevity.

### 4.1. TNTs for Enhancement of Soft and Hard Tissue Integration

The nanomaterials most thoroughly investigated for enhanced soft and hard tissue healing are titania nanotubes (TNTs) alone or in combination with other nanomaterials or biological agents. Balansudaram et al., in order to improve the bioactivity of TiO_2_ layer in titanium implants, utilized nanotubes to immobilize a segment of bone morphogenetic protein-2 (BMP-2), a protein proven to strongly induce bone formation. Due to the fact that BMP-2 is a large protein that cannot be directly adapted in TNTs, a smaller 20 amino acid sub-region called BMP-2 knuckle epitope was immobilized on TNT-manufactured implants through electrochemical anodization. In vitro analysis has shown increased osteoblast adhesion, rendering this an easy functionalization technique for enhanced bone implant quality [34]. In another in vitro study, Kodama and his team pre-loaded TiO_2_ nanotubes with synthetic HA through alternative immersion method (AIM) treatment and investigated apatite deposition in simulated body fluids model. Enhanced apatite formation was noted for this type of coating functionalization [35].

The effect of TNTs on soft tissue integration and human gingival fibroblast (HGF) behavior was also studied by Liu et al. in an in vitro experiment. TNTs were constructed in anodized Ti sheets and customized with bovine serum albumin (BSA), a protein promoting mineral deposition and affecting implant surface properties. Both TNT and TNT-BSA Ti implants displayed preservation of crestal bone and conductivity for soft tissue attachment with early adhesion and spreading of HGFs. BSA-treated implants additionally presented antibacterial properties as it was reported that HGFs adhered to the TNT-BSA implant surface before bacteria colonization, according to surface competition theory [36].

### 4.2. TNTs for Osteoporosis

To address the restrictions of osteoporotic environments, TNTs were loaded with anti-osteoporotic agents that affect bone metabolism such as bisphosphonates. Bisphosphonates are pyrophosphate analogs absorbed by osteoclasts during bone resorption, leading to apoptosis and eventually suppression of osteoclastic activity. Lee et al. manufactured titanium implants customized with TNTs loaded with ibandronate through anodic oxidation and thermal treatment and compared them to machine-turned and anodized heat-treated implants without the attachment of the anti-osteoporotic drug. It has to be highlighted that controlled continuous release of the drug from the TNTs was observed for the healing time of 4 weeks monitored during the experiment. These implants were placed in rats and removal torque analysis, microcomputerized tomography (μCT), and histologic analysis by Western Blot were performed 4 weeks after insertion. Higher removal torque values, increased bone density, and bone formation markers expression, respectively, were reported for ibandronate-functionalized implants. This surface modification seems to increase the biocompatibility of the implant and accelerate osseointegration and bone formation at the dynamic bone–implant interface [37]. A couple of years later, Shen and his team designed an implant doubly nano-functionalized for enhanced bone healing conditions. TNTs were created through anodic oxidation treatment in titanium implants and later customized with nano-HA on their walls. TNT-HA substrates serve as reservoirs for alendronate loading, a drug belonging to the bisphosphonate group. The rationale behind this double customization is to facilitate cell–material interaction and regulate the loading amount and controlled release of the drug. Surface analysis through SEM (Scanning Electron Microscopy), AFM (Atomic Force Microscopy) and XRD (X-ray Diffraction analysis) shows relative rough hydrophilic implant surfaces. In vitro tests demonstrate that the synergistic effect of alendronate and Ca ion release seems to positively contribute to osseointegration through the inhibition of osteoclast differentiation and the improvement of osteoblast activity. Complementary in vivo experiments in osteoporotic rabbits 3 months after implantation show higher interfacial strength and enhanced early local osseointegration and mechanical fixation [38]. Even though the systemic administration of bisphosphonates has been associated with significant side effects like osteonecrosis of the jaws (ONJ), the local use in dental implants is not contraindicated [39].

Except for anti-osteoporotic drugs, biological agents loaded into TNTs are also a viable strategy for the restoration of molecular functions in osteoporotic conditions. Zhang et al. fabricated titanium implants with nanotube arrays on their surface where recombinant human platelet-derived growth factor-BB (rhPDGF-BB) was immobilized. These implants were placed in ovariectomized rats, a classical postmenopausal osteoporotic animal model for experimentation. rhPDGF-BB is a key regulator in wound healing and tissue repair events with strong angiogenic potential. Slow release and sustained biological activity of the protein were noted affecting cellular events. Enhanced bone mesenchymal stem cell adhesion, proliferation, and differentiation, as well as rapid bone formation, were also detected [40]. Lee and his colleagues investigated the capability of TNTs loaded with N-acetyl-cysteine (NAC) to act as a potential drug delivery system in dental implants. NAC is a glutathione derivative and cysteine analog drug that serves as an anti-oxidant and ROS scavenger affecting oxidative stress. It is thought that NAC has the potential to reduce post-insertion inflammation and promote rapid bone formation. Surface analysis demonstrated optimized hydrophilic behavior and sustained controlled release for these NLN-Ti implants. In vivo measurements performed after insertion in rat mandibles have shown new bone formation and excellent osseointegration properties [41]. In a more recent study, Zhang et al. introduced a co-modified nanotube implant coating containing strontium (Sr) and lanthanum (La) elements for improved healing conditions in patients with insufficient osseointegration. Bone-promoting ions like strontium have been approved as a treatment option in patients with osteoporosis, aiming at the upregulation of osteoblast-related genes and increased bone repair. Lanthanum is a rare element improving bone density when used in low dosages with sustained release mode of action. The LSTN implant showcased superior osseointegration ability and increased cellular functions in vitro [42].

### 4.3. TNTs for Alleviation of Diabetes

Diabetes mellitus (DM) is another chronic disease associated with hyperglycemia due to insulin resistance (Type 1) or deficiency (Type 2). The disorder is characterized by delayed bone healing under hyperglycemic conditions due to impaired mineralization mechanisms and inhibition of osteoblast activity. These molecular events are related to oxidative stress and ROS overproduction, mediating inflammatory responses. Implant survival rates are lower for patients with Type 1 (80%) and Type 2 (90.5%) diabetes, while uncontrolled type 2 diabetes patients with dental implants are more common to suffer from peri-implantitis and marginal bone loss [43,44]. For this reason, Yang and his team investigated if the application of TiO_2_ nanotubes alleviates diabetic-induced osteogenic inhibition. Three types of Ti discs, mechanically polished, sandblasted and acid-etched (SLA), and TNT-treated were tested both in vitro and in vivo. TNT Ti discs displayed the best behavior as they reversed the overproduction of ROS and demonstrated antioxidant effects. The enhanced effect of TNTs can be attributed to the fact that their nano characteristics mimic bone and ECM components promoting signals for cell recognition of the surrounding microenvironment and biomaterial [12]. Gene delivery was also introduced to improve diabetic interference in healing processes. Lee and his team manufactured chitosan gold nanoparticles conjugated with PPARγ cDNA on titanium mini implants. This therapeutic gene affects the metabolism of glucose homeostasis and is involved in osteoblastic differentiation and bone remodeling procedures. In vitro and in vivo analyses have shown regional bone regeneration and improved osseointegration due to the reduction of inflammatory molecules. Chitosan-gold NPs can act as carriers for bioactive delivery on the grounds of easy DNA conjugation and evasion of immune detection [45]. Chitosan was also utilized for the creation of osteoconductive drug-loaded Ti coatings. Fang et al., in an in vitro experiment, incorporated Semaphorin 3A (Sema3A) in micro arc oxidized (MAO) Ti implants with the assistance of chitosan via silane glutaraldehyde coupling. Sema 3A is an osteoprotection molecule used as a therapeutic agent in bone diseases. When bound to chitosan films, the protein activity is maintained without cytotoxic events for almost two weeks and the release of the drug is controlled. This system did not seem to have an apparent cellular effect; only osteogenic-related gene expression and Ca apposition were found increased [46].

### 4.4. Other Nanomaterials

Except for titania nanotubes and chitosan, nanohydroxyapatite (nano-HA) coatings have been explored for enhancement of osseointegration. Yamada et al. fabricated micro-nano hybrid implants with nano-HA coatings through a combination of flame spray and low-temperature calcinations. Microroughened implants were coated with nanopolymorphic crystalline HA that presented nanoscale needle-like projections on the surface. These projections increased the surface-to-volume area by 70% when compared with non-coated microroughened surfaces. In vivo analysis has shown increased strength at bone–implant interface with reduced soft tissue intervention both at early and late stages and higher BIC and bone volume [47]. Zhao and his colleagues tried to incorporate magnesium (Mg) into nano-HA coatings in Ti implants treated with electrochemical deposition. Mg is a trace element of bone and teeth thought to contribute to bone metabolism. The element was implanted in hydroxyapatite before coating deposition. Promotion of osteogenesis was found in vitro and a slight influence in the improvement of osseointegration, mainly in early stages of bone healing, when studied in rabbit model [48].

Even though metal NPs are widely known for their antibacterial properties, which will be analyzed in the following paragraphs, some of them have the ability to improve implant integration. Gold nanoparticles can act as osteogenic agents presenting a positive effect on osteoblast differentiation. This property was exploited by Heo and his team to manufacture Ti implants coated with gold nanoparticles (GNPs). GNPs were immobilized in the silanized Ti implant surface via Au-S bonds. This surface functionalization stimulated cellular responses in vitro through upregulation in osteoblast-related genes and enhanced new bone formation especially in the bone–implant interface in vitro [49]. The use of silver nanoparticles has also been explored. In a study by Qiao et al., Ti implants were coated with silver nanoparticles through plasma ion implantation. This technique was selected to minimize the toxicity-related issues accompanying the inevitable unwanted release of silver ions to the tissues adjacent to the implant surface. When tested in vitro and in vivo, these implants displayed increased implant stability and enhanced bone formation, confirming the osteoconductive properties of Ag NPs [50].

Silicon plays an important role in metabolic bone processes, as it promotes collagen type 1 synthesis and osteoblast differentiation. Taking advantage of this property, Bartkowiak et al. synthesized a novel composite coating consisting of silica NPs entrapped in HA matrix, formulated under hydrothermal conditions. This biocompatible coating increased the bioactivity of the implant surface, inducing favorable mineralization of deposited bone matrix and accelerated bone healing [51]. In another study by Jo et al., SiNPs were utilized in order to tune the surface roughness of titanium-based implants. It was shown that SiNPs increase the microroughness of the implant improving its stability and creating osteopromotive conditions for improved bone-tissue growth [52]. In a study by Covarrubias et al., a hybrid nanostructured coating consisting of silica loaded with bioactive glass nanoparticles was deposited onto titanium implants. The silica coating nanotopography and the bioactivity of glass nanoparticles enhanced in vitro apatite formation [53]. A couple of years later, Vandamme et al. evaluated the osseointegration capacity of a titanium silica implant in vivo. Results showed that functionalization of titanium implants with SiO_2_ did not intervene with osseointegration, thus paving the road for exploring silica’s potential for drug release [54]. Frankenberger and his colleagues, seeking to enhance the biological behavior and bioactivity of polyetheretherketone (PEEK) implants, manufactured a coating consisting of nanocrystalline hydroxyapatite loaded into a silica matrix and interfacial composite layer (SPI). These implants were placed in adult rats and in vivo testing was performed. High bone-to-implant contact and pull-out forces were recorded for SPI implants, highlighting their mechanical strength [55].

Other nanomaterials used involve zirconia NPs and carbon derivatives. Frandsen and his team manufactured zirconium oxide nanotubes on zirconia implants via anodization. These nanostructures were proven to in vitro enhance initial cell response, namely adhesion and spreading and improve osteoblast growth [56]. Finally, Wang et al., aiming at inducing surface bioactive coatings in Ti alloy implants, introduced graphene oxide coatings fabricated by laser processing and chemical assembly. This modification increased the implant surface wettability and apatite formation towards new superior bone formation [57]. Table 1 presents several studies that focus on the use of nanomaterials for osseointegration improvement.

## 5. Immunomodulation Strategies

Several studies have focused on the implant adaptation, placement and assimilation, comparing the general behavior and performance of an implant with and without surface modification [58,59]. Implant surfaces play a fundamental role in the regulation of the inflammatory host response elicited after implantation. Two types of responses can take place, either fibrous tissue formation or bone-to-implant contact. Fibrous tissue formation leads to implant failure while bone formation to osseointegration, so immunomodulatory strategies have been developed in order to guide the molecular events towards bone apposition [33,60]. As described by Trindale and Albrektsson et al., osseointegration is a foreign body response that activates innate and acquired immune system mechanisms [61]. The main mechanism of innate immunity is inflammation. Early inflammatory events have been linked with macrophage activation, inflammatory cytokine production, and ROS generation. Macrophages are the basic regulator of these events and a crucial component in osseointegration dynamics. Dental implants can be accompanied by many molecules on their surfaces acting as antigens at the bone–implant interface, such as ions and nanoparticles that can pose a threat for the activation of excessive immune inflammatory responses. If inflammation is not resolved or is reactivated, there is a high risk of aseptic loosening, infection development, and finally bone loss and implant failure [62]. For this reason, immunomodulation strategies have been employed aiming at inducing well-controlled early inflammatory responses by controlling macrophage activity or incorporating anti-inflammatory drugs in implant surfaces [32,63].

### 5.1. Non-Biofouling Strategies

One of the first immunomodulation strategies involved the use of poly-ethylene-glycol (PEG) on the surface of Ti implants. PEG is a biocompatible polymer, able to passivate surfaces through protein and cell adhesion resistance. Kang et al. exploited PEG properties in order to manufacture a non-biofouling, bioinert titanium surface. His team coated titanium substrates with PEG methacrylate (pPEGMA) via SAM polymerization. pPEGMA films were additionally functionalized with BMP-2 to enhance the bioactivity of the surface. In vitro experiments have shown excellent non-biofouling and simultaneous osteoconductive properties for these dual functionalized Ti implants [64].

The surface of the implant material alone is significant in the induction of immune reactions. Nanoscale architectures are shown to decrease immune responses due to their ability to mimic the natural molecular and cellular environments. In an in vitro experiment, Smith and her team investigated short- and long-term immune cell reactions on TNTs. Analysis has shown a decrease in monocyte, macrophage, and neutrophil functionality and reduced stimulation of immune responses [65]. A couple of years later, Neacsu et al. investigated the potential of TNTs in the regulation of inflammatory activity. TNTs were fabricated via electrochemical anodization in Ti foils and cell culture, ELISA assay determination, and immunofluorescence staining were performed. The results showed that TNTs can attenuate the macrophage inflammatory response through suppression of mitogen-activated protein kinase (MAPK) and nuclear factor kappa-light-chain-enhancer of activated B cells (NF-κΒ) signaling pathways. Those two pathways are crucial for the initiation of macrophage-related inflammation and their suppression prompts a reduced stimulation of the immune system [66]. Nanopores were also studied for their bioinertness. Gulati et al. fabricated nanopores via electrochemical anodization on microgrooved titanium implants and investigated the cellular reactions to these dual nano-micro modified surfaces. The proliferation of macrophages, as well as their alignment, was found reduced, while osteoblast and fibroblast activity was increased [67]. In a recent study, Li et al. prepared three types of nanoarrays to investigate the effect of surface morphology in healing phenomena. Nanorod arrays with a diameter of 45 nm and 60 nm as well as nanocone arrays were constructed on titanium. It was shown that nanorods with 60 nm diameter promoted osteogenic differentiation of BMSCs and regulated macrophage polarization to the M2 phase. M2 phase macrophages are associated with anti-inflammatory, repair, and regeneration events while M1 macrophages with inflammation phenomena. The polarization from M1 to M2 phase is crucial in wound healing procedures [68].

To control macrophage activity, a variety of different coatings have been proposed. Su et al. incorporated a graphene oxide (GO) coating through dopamine on pure titanium surfaces. In vitro experiments showed that GO manipulated the polarization of macrophages and the expression of inflammatory cytokines, thus displaying immunomodulatory effects in osteogenesis [69]. In a study by Li et al., a thermosensitive hydrogel on anodized titanium surfaces was exploited in order to control immune responses. This hydrogel was composed of hydroxypropyl methylcellulose, chitosan, and glycerin and has the ability to identify changes in temperature. At normal temperatures, it is in sol state causing macrophages to polarize towards the M2 phase, promoting tissue repair, while at high temperatures in bacteria-infiltrated tissues it transforms to a gel state polarizing macrophages to the M1 phase [70]. In another study by Chen et al., curcumin was loaded through polydopamine (PDA) onto copper-bearing titanium alloys (Cu-Ti). Curcumin displays anti-inflammatory and antibacterial properties and can regulate macrophage polarization towards the M2 phase. In vitro analysis indicated that these alloys could implement immune regulation of macrophages through control of their polar differentiation [71]. Recently, Liu and his team manufactured a metal phenolic network (MPN) nanocoating consisting of tannic acid and strontium on Ti substrates. Results of in vitro and in vivo analysis showed that MPN coatings created a favorable osteoimmune environment, transforming the microenvironment from pro-inflammatory to pro-healing state by regulating the polarization of macrophages to the M2 phase [72].

### 5.2. Anti-Inflammatory Drug Loading

Nanomaterials have also the ability to load anti-inflammatory drugs on their core or their surface to provoke certain interactions. Doadrio et al. utilized TNTs as a drug delivery system to bind ibuprofen, an anti-inflammatory drug. This study was mostly performed to assess the pharmacokinetics of the drug and prove the ability of TNTs to act as an intelligent nanomaterial. The release of the drug was found constant and independent of the concentration [73]. Taking advantage of Doadrio’s findings, Shen and his team manufactured an implant for sustained release of dexamethasone (DEX). DEX is a glucocorticoid hormone that can positively affect osteoblastic differentiation of MSCs and regulate signaling pathways and macrophage activity. For this purpose, TNTs were created via anodization, filled with the anti-inflammatory drug, and then covered by chitosan multilayer films. Osteoblast and macrophage cells were cultivated on those surfaces and the reactions elicited were compared to a control group. Proliferation and differentiation of osteoblasts were enhanced, while macrophages displayed suppressed production of nitric oxide (NO) and pro-inflammatory cytokines, rendering TNT-Cht implants loaded with DEX a viable approach for immunomodulation [74]. DEX-loading was also exploited in implants functionalized with silica NPs. Luo and his team fabricated mesoporous silica nanoparticles (MSNs) on titanium implants via “sol-gel” method, which were later loaded with DEX. They were based on the fact that DEX seems to induce macrophage polarization towards M2 direction. M2 polarized macrophages promote new bone formation, whereas M1 are linked with inflammatory responses. In vitro experiments have shown favorable osteogenesis but dose-dependent toxicity. These DEX-loaded implants demonstrated potential to modulate immune responses [75]. Biopolymers, like polylactic-co-glycolic acid (PLGA), have also been explored as novel drug delivery systems. PLGA is a biocompatible and biodegradable polymer with the ability to be used as a drug loading material. Wei et al. constructed Ti implants with PLGA nanofiber coatings via electrospinning. The PLGA nano-coating was additionally loaded with aspirin, a non-steroidal anti-inflammatory drug (NSAID). The release of the drug was stable and could be sustained for up to two months. In vitro analysis showed inhibition of M1 polarization and increased proliferation and differentiation of MSCs to osteoblasts, while in vivo experiments in rats demonstrated enhanced osseointegration [76]. The same model was utilized by You and his team in 3D printed Ti alloy implants (Ti-6Al-4V). A PLGA/aspirin coating was superimposed on the implant surface and in vitro analysis was performed. Like the previous study, enhanced M2 gene and protein expression was noted confirming the immunomodulatory abilities of this nano-based surface modification. In vivo evaluation through push-out tests, μCT, and histological analysis demonstrated the superiority of the PLGA/aspirin functionalized surface [77].

Another strategy recently explored is the incorporation of extracellular vesicles into titanium nanotube (TNT) decorated Ti surfaces. Extracellular vesicles (EVs) participate in cellular communication and tissue engineering procedures and have the ability to influence cellular responses and alleviate inflammation. Taking advantage of their properties, Zhao et al. created hybrid coatings bearing MSC-derived exosomes on TNTs. This double-layer customization of TNTs included a first internal layer of MSC-derived exosomes adhered in polydopamine serving as a pro-osteogenic factor and a second external layer of 3-day osteogenically differentiated MSC-derived exosomes loaded in carboxymethyl chitosan hydrogel for MSC attraction. In vitro analysis confirmed the coating’s immunomodulatory role for macrophages [78]. In another study, Jayasree et al. incorporated microvesicles (MVs) isolated from human gingival fibroblasts (hGF) onto titania nanotubes anodized implants. Microvesicles are a type of extracellular vesicle shown to modulate angiogenesis and inflammation. MV-releasing nanotubular implants demonstrated a controlled local release of MVs for up to 7 days and reduced pro-inflammatory cytokine production in keratinocytes. This subsequently resulted in enhanced tissue integration [79]. Some of the studies that deal with nanomaterials for immunomodulation are shown in Table 2.

## 6. Prevention of Peri-Implantitis

Infectious complications, like peri-implantitis and peri-implant mucositis, can disrupt the process of osseointegration and even lead to implant loss [78,80]. Peri-implantitis is a microbial biofilm-mediated pathological entity, characterized by inflammation of the peri-implant mucosa and progressive loss of supporting bone [81,82]. The onset and progression of the disease follow an unpredictable, non-linear, and accelerating pattern [83]. Peri-implantitis is observed in 15%–57% of patients and 8%–28% of implants, depending on the case definition applied in each study [84].

The oral microbiome consists of approximately 700 species, forming an organized community attached to surfaces, called biofilm. The microorganisms participating in the biofilm are protected by an exopolysaccharide (EPS) matrix constructed by proteins, lipids, and extracellular DNA. The change in the microbial flora from Gram-positive, non-motile, and aerobic bacteria to Gram-negative, motile, and anaerobic bacteria is associated with peri-implant diseases and implant failure. The species contributing to infectious complications in implants are Fusobacterium nucleatum, Aggregatibacter actinomycetemcomitans (Aa), Porphyromonas gingivalis, Staphylococcus aureus, Streptococcus mutans, Candida albicans, Escherichia coli, Streptococcus gordonii, and Streptococcus sanguinis [80,85].

To reduce microbial infections, modifications of the implant surfaces were introduced with the incorporation of antibiotics and nanoparticles. Some of the nanoparticles have inherent antimicrobial properties due to their roughness or composition and can even be loaded with additional anti-microbial and anti-biofilm formation substances [80].

### 6.1. NPs with Inherent Antibacterial Properties

Many nanomaterials, like titania nanotubes and metal NPs, demonstrate inherent antibacterial properties [86,87,88]. Puckett and her team tested surface roughness in accordance with bacterial adhesion on Ti orthopedic implants this time. This in vitro study has shown that nanorough Ti surfaces, created by electron beam evaporation, displayed decreased bacterial adhesion especially of *S. aureus*, *S. epidermidis*, and *P. aeruginosa*, while nanotubular and nanorough Ti surfaces manufactured through anodization presented an increase in bacterial attachment. The degree of surface roughness and the fabrication method utilized can determine the behavior of the surface against bactericidal attacks [89]. Taking advantage of the antibacterial property of silver, Cao et al. embedded AgNPs on Ti implants through one-step plasma immersion ion implantation. SEM analysis shows NPs with average sizes of 5 nm and 8 nm. In vitro experiments showcased inhibition of *S. aureus* and *E. coli* growth and enhanced antibacterial activity of the surface due to micro-galvanic effects. However, the physicochemical characteristics of AgNPs seem to affect the cytotoxicity of the system [90]. Seeking to strike a balance between antibacterial effects and good biocompatibility with minimized toxicity, Zhu et al. immobilized AgNPs on SLA-treated implants with the plasma immersion ion implantation technique. In vitro experiments have shown increased anti-bacterial activity against gram-positive *S. aureus* and gram-negative *F. nucleatum*, with *F. nucleatum* being more susceptible due to its less rigid cell wall structure. This effect was found independent of silver NPs release [91]. A couple of years later, Lampe and his colleagues used the same manufacturing method as the previous studies to anchor silver NPs on Ti implants. A 64.6% antibacterial effect was noted for the nanoparticle-covered samples, implying inhibition of peri-implant inflammation [92]. The same principle was employed by Liu and his team, by manufacturing a novel nanocomposite layer of silver-containing hydroxyapatite in Ti alloy implants via laser processing. SEM and XRD analysis shows a 200 μm layer of Ag-HA on the interface fused with the substrate surface. This nanocomposite demonstrates bacterial inhibition for a percentage of 2% silver but cytotoxicity when this percentage is increased [93]. A study by Gosau et al. investigated the antibacterial effects of silver (Ag), copper (Cu), and bismuth (Bi) on Ti discs. Nanocrystalline Ag, Cu, and Bi were used through pulsed-magnetro sputtering to coat Ti discs. Atomic force microscopy measures have shown increased surface roughness for Bi-coated discs, while cytotoxicity assays displayed strong cytotoxic effects for Cu implants. Overall, all three coatings resulted in favorable anti-bacterial effects, but only Si and Bi seem to be viable options for prevention of peri-implant infections due to toxicity issues with copper [94]. Hameed et al. studied, in an in vitro model, copper NP and copper NP-doped HA coatings in Ti alloy dental implants. Disk diffusion tests and broth culture analysis demonstrated enhanced antibacterial effect against *P. gingivalis*. However, as the previously mentioned study indicated, they did not examine and corroborate the biocompatibility of the nanomaterial [95]. Except for metal nanoparticles, polymers also possess intrinsic antibacterial properties. Liu et al. implemented a polydopamine (PDA) coating on zirconia implants, seeking to enhance soft tissue integration in zirconia implants and reduce bacteria colonization. In vitro analysis demonstrated increased cell adhesion and proliferation of hGF, as well as decreased bacterial adhesion [96].

### 6.2. NPs Loaded with Drugs

The ability of titania nanotubes to load and release potent antibacterial and antibiotic agents, as well as to synergistically act with other nanomaterials, is also exploited for the prevention of peri-implant infections both in the early healing period and for the maintenance of peri-implant health [19]. Zhao et al. incorporated AgNPs on the inner walls of TNTs through AgNO_3_ immersion and UV irradiation to enhance the anti-bacterial properties of the substrate. The size and the amount of silver nanoparticles are modulated by AgNO_3_ concentration and immersion time to counteract cytotoxic effects. In vivo analysis showed that AgNP-functionalized TNT surfaces can kill planktonic bacteria for the first days after surgery and inhibit bacterial adhesion for 30 days, thus preventing post-operative and even late infectious complications [97]. Following the same idea, Huo et al. immobilized zinc nanoparticles in TNTs via anodization and hydrothermal treatment. The Zn amount is adjustable to avoid cytotoxic events, just like the Ag in the previous experiment, by tuning the fabrication parameters. Good intrinsic antibacterial properties with simultaneous favorable soft and hard tissue integration were recognized in in vitro analysis for this system [98]. Wang et al., in order to enhance the implant bioactivity, fabricated a graphdiyne (GDY) composite TiO_2_ nanofiber coating. TiO_2_ is a photocatalytic material that has the ability to produce ROS under UV irradiation to destroy bacterial species, while GDY, with its stability, biocompatibility, and superior electrical conductivity, enhances the catalytic effect of metals. It has to be highlighted that graphene is an allotrope of GDY with osteoconductive properties. In vitro and in vivo experiments have shown increased photocatalysis and prolonged antibacterial ability, especially against methicillin-resistant staphylococcus aureus (MRSA). ROS release from this system prevented the formation of biofilm [99].

The combination of TNTs for drug elution with polymers is a common strategy for targeted sustained and controlled anti-inflammatory and antibiotic drug delivery. Biopolymer coatings have inherent antibacterial properties, the ability to control the release of the desired drug, and the capacity to promote the bioactivity of the surface they are attached to. Gulati and his team utilized polymer coatings to regulate the release of indomethacin from TNT-modified implants. A titania layer of nanotube structures was fabricated on Ti implants through electrochemical anodization, where the water-soluble anti-inflammatory drug indomethacin was incorporated. The system was covered by a thin polymer film (chitosan or PLGA) applied via a dip-coating process. In vitro experiments have shown extended drug release properties, favorable bone cell adhesion, and improved anti-bacterial properties for this device. Reduced burst release and extended overall release from 4 to 30 days was registered, while the thickness of the polymer seems to regulate the drug release characteristics of the system [100]. The same principle was applied by Kumeria et al. towards the creation of multifunctional implant devices with a triple role: controlled drug release, improved osseointegration, and antibacterial properties. With the assistance of drug-encapsulating micelles, TNTs were loaded with gentamicin, a popular antibiotic, and later covered with antibacterial polymers, either chitosan or PLGA. Gentamicin was mostly utilized as a drug model for the investigation of pharmacokinetics for this system. Long-term and improved anti-bacterial properties were found for the device, with prevention of biofilm formation especially from chitosan-coated implants [101]. A couple of years later, Baghdan et al. utilized polylactic-co-glycolic acid (PLGA) to coat titanium discs and then loaded them with norfloxacin, a broad-spectrum antibiotic. PLGA is a biodegradable polymer with the ability to regulate controlled drug release. In vitro analysis showed 99.83% reduction in bacterial colonies in PLGA-drug discs. Additionally, a biphasic release profile was achieved, with an initial burst release dose and a later maintenance dose [102]. Bacteria in biofilm communities have the ability to develop resistance to certain antibiotics through gene transfer. To resolve this problem while retaining the anti-bacterial properties, Ma et al. introduced antimicrobial peptides (AMPs) on the surface of TNT-treated implants. Cationic AMPs present low toxicity, little possibility to develop resistance, and good antibacterial properties. For this experiment, TNT self-organized structures were created through anodization on Ti foils, and HHC-36, a prominent member of the AMP family, was incorporated into them via the vacuum-assisted adsorption technique. In vitro analysis followed, where it was found that AMP-loaded nanotubular architectures can reduce gram-positive bacterium *S. aureus* levels and effectively diminish bacterial adhesion on the implant surface. The crystallinity of the TiO_2_ nanotubes significantly affects the release profile of the drug but without influencing the overall efficacy of the agent [103]. In a recent study, Srivastava et al. introduced a macroporous titanium matrix filled with mesoporous silica (TiSO_2_) capped by crosslinked chitosan, which has pH-responsive and antibacterial properties. To further enhance antibacterial activity, chlorhexidine (CHX) was loaded to the system. Chitosan regulated controlled release of CHX and showed reduced numbers of bacterial growth compared to the uncoated Ti/SiO_2_ sample, especially against *S. sobrinus* and *F. nucleatum* [104].

### 6.3. Chitosan Hybrid Coatings

The intrinsic antibacterial abilities of polymers have also been coupled with those of metal nanoparticles. Cheng et al. deposited silver NPs on catechol-containing chitosan (CACS) coatings to prevent bacterial adhesion on Ti implants. Catechol acts as a reductive agent for the in situ synthesis of Ag ions. In vitro analysis involved the use of inhibition zone test, live/dead bacterial staining assay, and spread plate method, confirming the anti-bacterial properties of the system, both against gram-positive and gram-negative bacteria. The growth of *S. aureus* and *E. coli* was inhibited, with S. aureus being more susceptible towards the antibacterial device. Toxicity was found negligible in MTT assay evaluation [105]. Mishra and his team encapsulated polyvinyl alcohol (PVA)-capped AgNPs into a chitosan matrix covering Ti implants. Colloidal sols of PVA-covered AgNPs were created with the microwave approach and later dispersed in an aqueous solution of chitosan medium. The bionanocomposite was deposited onto the implant surface with spread casting and subsequent solvent evaporation. The use of a capping agent can be attributed to the improvement in material performance and minimization of hazardous by-products they offer. In vitro experiments demonstrate better functional properties and enhanced bactericidal activity against *S. aureus* and *E. coli* [106]. Song et al., investigating orthopedic implant modifications, introduced gelatin nanosphere antibiotic-loaded substrates. These gelatin nanospheres were encapsulated into a chitosan matrix and bound with vancomycin and moxifloxacin through electrophoretic deposition. Homogeneous distribution of gelatin nanospheres into the chitosan matrix was described, while the gelatin nanosphere/chitosan ratio determined surface characteristics such as roughness and wettability. Zone inhibition tests have shown inhibition of bacterial growth for both antibiotics. This double antibiotic-loaded substrate displayed control over the release of each substance [107]. Choi et al. combined silver nanoparticles with polydopamine (PDA), a biopolymer, to form an antibacterial coating candidate. This coating was created by immersing pure titanium in dopamine/HCl buffer solution for 24 h, achieving uniform silver nanoparticle distribution in the PDA matrix. In vitro experiments showed less bacteria colonization in Ag/PDA-treated implants when compared with uncoated titanium surfaces, and bacterial growth was found retarded in bacterial growth curves for *S. mutans* and *P. gingivalis* [108]. Finally, in a study by Palla-Rubio et al., the antibacterial properties of chitosan were combined with silica osteoinductivity in a sol-gel manufactured hybrid coating. In vitro analysis demonstrated effective silicon release from the hybrid coatings, enhancing bone formation and increased antibacterial properties for 5% to 10% of chitosan [109].

### 6.4. Other Nanomaterials

Silica NPs have been explored alone or in combination with other nanomaterials for the induction of antibacterial properties to dental implants. Xu and his team, taking advantage of the drug-loading capacity of mesoporous silica nanoparticles (MSNs), incorporated the cationic antiseptic agent octenidine dihydrochloride (OCT) into the coating. The MSNs were constructed via electrophoretic-enhanced micro arc oxidation technique, and then the encapsulation of the drug followed. Inhibition of bacterial adhesion was noted, especially for *S. mutans* and *E. coli* [110]. In a study by Li et al., silica membranes were used in combination with other nanomaterials to form an antibacterial bioplatform. A hybrid coating consisting of monodispersed polystyrene-acrylic acid (PSA) nanoparticles, zinc oxide (ZnO), a silica film on the outside, and N-halamine polymer labeling was tested for its biocompatibility and anti-microbial properties. In vitro analysis displayed excellent anti-bacterial activity against *P. aeruginosa*, *E. coli*, and *S. aureus* with no obvious cytotoxicity [111]. Kulshrestha et al. manufactured a graphene zinc oxide coating and investigated its effect on biofilm formation on acrylic teeth surfaces. Microscopic and anti-biofilm assay evaluations showed a reduction in biofilm deposition without apparent cytotoxicity, implying that this nanocomposite can have applications as a promising anti-microbial implant coating [112].

Finally, liposomes have been investigated as potential drug or bioactive molecule carriers. De Leo et al. investigated two different liposome coatings: one supported vesicular layer created by liposome adhesion on the passivated Ti substrate and one covalently bonded vesicular layer deposited on the functionalized Ti surface. The study focused on the assessment of the ability of the system to stay anchored and stable on the implant surface. Photoluminescence spectroscopy and AFM showed efficient attachment of liposomes on the Ti surface. This has enormous importance because the system can be utilized for the incorporation of various moieties with different polarities such as antibiotics, anti-inflammatory drugs, and protein-like growth factors. They can simultaneously encapsulate hydrophilic, hydrophobic, and amphiphilic materials [113]. Table 3 highlights important information obtained from studies focusing on nanomaterials for the prevention of peri-implantitis.

## 7. Corrosion Resistance

Another area where nanomodifications were introduced was the formation of dental implants with good behavior against corrosion. Corrosion can be distinguished in mechanical, chemical/electrochemical, and tribocorrosion. Mechanical corrosion is induced during surgical preparation and implant placement, as well as functional loading. Electrochemical corrosion is associated with reduced pH levels from acidic substances and microbial metabolites, while tribocorrosion is a combination of wear and fretting with chemical phenomena [114]. These factors affect the nanoscale amorphous TiO_2_ layer that, under physiological conditions, has the ability to act as a barrier preventing corrosion and subsequent ion release. When the conditions applied change and the implant is placed in vivo, the reduced pH and the mechanical forces exerted upon its surface under loading lead to partial or complete dissolution of the metal layer. These new conditions make the implant more vulnerable to electrochemical reactions taking place between its surface and oral fluids. Ion release from oxidized implant surfaces has been linked with the onset and progression of peri-implant bone loss and is considered a risk factor for peri-implantitis, even though this relationship has not been confirmed [114]. Additionally, cleaning and disinfection of implant surfaces during maintenance seem to constitute a risk for tribocorrosion [115].

Indira et al. manufactured TNT assays through electrochemical anodization and loaded them with zirconia (Zr) ions by dip-coating method in Ti implants for orthopedic applications. In vitro analysis has shown that HA has grown over Zr ions, while enhanced corrosion resistance was demonstrated by the implant system when immersed into Hank’s solution [116]. Al-Saady et al. constructed titanium oxide nanotube substrates via electrochemical anodized treatment and immersed them in SBF. SEM and AFM testing the titanium oxide layer indicated that increased voltage application resulted in nanotubes with higher corrosion resistance. The anodizing process seems to affect the corrosion behavior of titanium oxide nanotubes through alterations in morphology and surface properties of the oxide layer. Nevertheless, the addition of nanotubes in titanium improved its corrosion resistance [117]. In a recent study by Azari et al., the titanium dioxide (TiO_2_) layer was utilized as an intermediate layer between the HA coating and the Ti6Al4V substrate, due to the fact that HA coatings are fairly unstable. Both TiO_2_ and HA layers were prepared through the sol-gel method, creating a low-thickness coating. Overall, improved stability, adhesion, hardness, and tribological performance were recorded. When immersed in SBF, the TiO_2_ intermediate layer reduced the corrosion current by 65% and enhanced the corrosion resistance of the substrate [118].

Silica compounds and nanoparticles, except for their excellent ability to encapsulate drugs, can also contribute to the mechanical resistance of implant devices. Shen and her colleagues customized Ti surfaces with nano silicon nitride (Si_3_N_4_) particles through micro-arc oxidation (MAO) treatment. These surfaces exhibited excellent osteoinduction and angiogenetic properties, enhancing the initial healing stages. Regarding corrosion, tendency and rate were found significantly diminished, dependent on Si_3_N_4_ concentration [119]. In another study, Afrouzian et al. utilized silica as a ceramic coating on the surface of Ti6Al4V surfaces with the intent to increase hardness and wear resistance. Focusing on orthopedics, improved tribological performance was shown for these types of implants [120]. A pilot study by Hsu et al. investigated the potential of silicon carbide (SiC) attached on anodized titanium dioxide nanotubes (ATO), due to the material’s biocompatibility, strength, and corrosion resistance. To confirm this hypothesis, the SiC-loaded nanotubes were exposed to NaCl solution and bacteria incubation. Improved corrosion resistance was shown for SiC-coated ATO when compared with bare ATO [121].

Hybrid coatings have also been explored in order to address a variety of features in dental implants, with corrosion resistance being one of them. In order to improve both bioactivity and corrosion resistance, Harb et al. created a hybrid organic-inorganic coating deposited on Ti6Al4V substrates. The ability of PMMA nanocomposites for corrosion protection and durability was combined with titanium’s and zirconium’s bioactivity. Osseointegration ability was further enhanced by the addition of hydroxyapatite (HA) and β-tricalcium phosphate (β-TCP). PMMA-TiO_2_ and PMMA-ZrO_2_ doped with HA and β-TCP presented excellent bone integration and protection from corrosion wear in SBF. Especially PMMA-TiO_2_-β-TCP coatings presented low-frequency independence modulus unchanged for 21 days [122]. Kazemi and her colleagues investigated a hybrid coating consisting of titanium nitride (TiN) and hydroxyapatite (HA) deposited on Ti6Al4V alloy. This hybrid coating showed increased corrosion resistance [123]. In another study, Aydin et al. decorated titanium dioxide nanotubes with zinc oxide (ZnO) nanorods and silver nanoparticles (AgNPs) to enhance antibacterial activity. Except for the enhanced antifungal activity of ZnO-TiO_2_-NTs, high resistance value was found for this material when immersed in SBF for 7 days. This high corrosion performance is linked with the blockage of nanotube channels from ZnO, thus interrupting contact of the metal with the solution [124]. Xia et al. incorporated carbon and copper nanoparticles in medical titanium for enhancement of corrosion resistance. The co-implantation of C/Cu was performed with the same manufacturing method as the previous experiment. In vitro experiments displayed improved mechanical properties and reduction of free copper ions. The Cu ion release was regulated by the galvanic corrosion effect of the system, with no additional cytotoxicity induced [125].

In an in vitro study by Zheng and his team, a zirconium film was deposited onto a TiNi alloy substrate via plasma immersion ion implantation and deposition. Reduced Ni ion release and improved corrosion resistance were noted for Zr-coated substrates [126]. Yusuf et al. investigated a novel nano zirconia implant consisting of partially stabilized zirconia (PSZ) doped with magnesia (MgO). Biodegradation tests in SBF showed degradation resistance was dependent on the concentration of MgO in PSZ doping, with high Mg-PSZ exhibiting greater degradation resistance [127].

Finally, Zaher et al. tested a calcium phosphate coating (CaPO4) consisting of amorphous calcium phosphate nanoparticles (ACP-NPs) in a simulated saliva solution while adding essential oils, like cumin, thyme, and coriander. This study demonstrated that the titanium surface response is not soleley dependent on the morphology but other factors, such as the solution medium, may influence implant stability [128]. Table 4 summarizes the main points of studies that are related to nanomaterials for corrosion resistance.

## 8. Future Perspectives

Nanomedicine opened new frontiers in the rapidly advancing field of dental implantology. Nano-based modifications were introduced in implant surfaces in order to control cell and molecular events and have an impact on soft and hard tissue integration. Their basic aim was to navigate healing towards osseointegration over fibrous encapsulation through enhanced nanotopography. This is possible only when the nanomaterial applied has the ability to be incorporated in the human body without eliciting immune responses that may lead to rejection of the foreign device. Therefore, non-biofouling is crucial for nano-based implant modification strategies. The incorporation of additional biological agents and drugs in nanocarriers has an impact both in early healing events and in long-term implant longevity and functionality. Titania nanotubes and silica nanoparticles play a leading role as drug, biologic, and gene carriers, with biopolymers regulating the release profile of the incorporated agent. The inherent antibacterial properties of many nanomaterials, such as metal nanoparticles, have a huge impact on the prevention of peri-implantitis, an inflammatory disease threatening implant survival. Finally, the enhancement of corrosion resistance is pivotal for the protection of peri-implant tissues from nanoparticle infiltration and subsequent potential inflammation induction.

Finding the ideal surface combining most of the aforementioned properties, without generating cytotoxicity, is going to be a challenge for researchers for the next few years. There are still several research gaps due to limitations in material science advancements and understanding. The ultimate goal is to be able to predict and modulate cell responses. One of the areas where the focus should be directed is in the regulation of local elution of drugs. The studies mentioned investigate only the burst and short-term release of the incorporated drug, restricting their action for only a period of 1–2 months. The challenge is to create nanodecorated surfaces carrying agents with a long-term effect on peri-implant tissue health. A potential implementation of such a strategy would involve the introduction of smart implants with the ability to respond to internal or external stimuli, thus modulating the release of the desired agent when appropriate. Another field where nanomedicine could be of assistance would be the use of nanomaterials in medically compromised patients with osteoporosis, diabetes, and advanced age, where the healing mechanisms are impaired. Additive manufacturing could play a fundamental role in fabricating patient-specific implants. Tailored 3D printed implants can be customized according to the patients’ conditions and rehabilitation requirements. The surface characteristics and nanomaterial applications can be tuned to acquire the desired clinical effect. However, given that 3D printing is a relatively new technology, its use is limited due to its high cost and time-consuming nature.

The authors have to highlight that most of the studies mentioned in the previous chapters are in vitro studies, and further in vivo testing and safety assessments are required before moving into clinical trial experimentation. The implications of sterilization, packaging, handling, implantation, and operation on the nano-modified surface should also be considered before widespread commercialization [19]. There are many more steps to be taken in order to permanently link the fate of implantology with nanotechnology.

To conclude, it is evident that several nanomaterials have been proposed for nano-based implant surface modifications due to their advantageous properties. These mainly include the enhancement and preservation of osseointegration, alongside the inhibition of peri-implant disease. Even though these modifications have been extensively studied in in vitro experiments showing great promise, there is still a long way to go until clinical use. With the goal of achieving a clinical translation, it is crucial that in vivo experiments in suitable animal models are performed as well as studies that will investigate the capabilities of large-scale production of these nanomaterials.

## Figures and Tables

**Table 1:** Studies with nanomaterials for osseointegration improvement. Abbreviations: TNTs: Titania Nanotubes; BMP-2: Bone Morphogenetic Protein-2; HA: Hydroxyapatite; BIC: Bone to Implant Contact; Mg: Magnesium; NAC: N-acetyl-cysteine; BSA: Bovine Serum Albumin; rhPDGF-BB: recombinant human Platelet Derived Growth Factor-BB; MSC: Mesenchymal Stem Cells; Ca: Calcium; AgNPs: Silver Nanoparticles; GNP: Gold Nanoparticle; SiNPs: Silica Nanoparticles; Au: Gold; GO: Graphene Oxide; Sr: Strontium; La: Lanthanum; ROS: Reactive Oxygen species.

Studies with Nanomaterials for Osseointegration Improvement			
Study	Method	Nanomaterial	Result
Yang et al., 2020 [12]	In vitro and in vivo	TNTs	Reversed overproduction of ROS, antioxidant effect
Balasundaram et al., 2007 [34]	In vitro	TNTs loaded with BMP-2	Increased osteoblast adhesion
Kodama et al., 2009 [35]	In vitro	TNTs loaded with synthetic HA	Enhanced BIC and apatite formation
Liu et al., 2014 [36]	In vitro	TNTs loaded with BSA	Preservation of crestal bone, conductivity for soft tissue attachment, antibacterial properties
6Lee et al., 2011 [37]	In vitro and In vivo	TNTs loaded with ibandronate	Higher removal torque values, increased bone density and bone formation markers expression
Shen et al., 2016 [38]	In vitro and in vivo	TNTs and HA loaded with alendronate	In vitro inhibition of osteoclast differentiation and the improvement of osteoblast activity and in vivo early local osseointegration and mechanical fixation
Zhang et al., 2014 [40]	In vitro and In vivo	TNTs loaded with rhPDGF-BB	Enhanced MSC adhesion, proliferation and differentiation, rapid bone formation
Lee et al., 2013 [41]	In vivo	TNTs loaded with NAC peptide	New bone formation, excellent osseointegration
Zhang et al., 2020 [42]	In vitro	TNTs loaded with Sr-La	Superior osseointegration ability and increased cellular functions
Lee et al., 2017 [45]	In vitro and in vivo	Chitosan-Au NPs with PRAγ cDNA	Regional bone regeneration and improved osseointegration
Fang et al., 2014 [46]	In vitro	Chitosan loaded with Sema 3A	Higher osteogenic gene expression and Ca apposition
Yamada et al., 2012 [47]	In vivo	Nano-HA on microroughened implants	Increased strength at bone implant interface, higher BIC and bone volume
Zhao et al., 2011 [48]	In vitro and in vivo	Nano-HA Mg doped	In vitro promotion of osteogenesis and in vivo improvement of osseointegration
Heo et al., 2016 [49]	In vitro and in vivo	GNP coating	In vitro stimulated cellular responses and in vivo enhance new bone formation
Qiao et al., 2015 [50]	In vitro and in vivo	AgNPs	Increased implant stability and enhanced bone formation
Bartkowiak et al., 2018 [51]	In vitro	SiNPs on HA treated implants	Favorable mineralization of deposited bone matrix and accelerated bone healing
Jo et al., 2017 [52]	In vitro and in vivo	SiNPs	Increase microroughness, osteopromotive conditions
Covarrubias et al., 2016 [53]	In vitro and in vivo	Nanoporous silica coating loaded with bioactive glass nanoparticles (nBG/NSC) on Ti implants	Accelerate the formation of bone tissue in the periphery of the implant after 3 weeks of implantation.
Vandamme et al., 2020 [54]	In vivo	Mesoporous SiO_2_ customization on Ti implants	Does not seem to compromise the osseointegration process.
Frankenberger et al., 2021 [55]	In vivo	Nanocrystalline hydroxyapatite (ncHA) embedded in a silica matrix and interfacial composite layer (SPI) on PEEK implants	Higher bone to implant contact (BIC) and pull-out tests revealed higher pull-out forces.
Frandsen et al., 2011 [56]	In vitro	Zirconia nanotubes	Enhanced cell adhesion and spreading and improved osteoblast growth
Wang et al., 2019 [57]	In vitro	GO	Increased surface wettability and apatite formation

**Table 2:** Studies with nanomaterials for immunomodulation. Abbreviations: PEG: Poly-ethylene-glycol; BMP-2: Bone Morphogenetic Protein-2; Ti: Titanium; TNTs: Titania Nanotubes; MAPK: Mitogen Activated Protein Kinase; NF-κΒ: kappa-light-chain-enhancer of activated B cells; MSNs: Mesoporous Silica Nanoparticles; DEX: Dexamethasone; Cht: Chitosan; PLGA: Polylactic-co-glycolic acid.

Studies with Nanomaterials for Immunomodulation			
Study	Method	Nanomaterial	Result
Kang et al., 2010 [64]	In vitro	PEG and BMP-2 on Ti implants	Non-biofouling and simultaneous osteoconductive properties.
Smith et al., 2013 [65]	In vitro	TNTs	Decrease in monocyte, macrophage and neutrophil functionality and reduced stimulation of immune responses.
Neascu et al., 2015 [66]	In vitro	TNTs	Suppression of MAPK and NF-κB pathways, potential mechanism for anti-inflammatory activity.
Gulati et al., 2018 [67]	In vitro	nanopores	Reduced proliferation of macrophages, increased osteoblast and fibroblast activity.
Li et al., 2023 [68]	In vitro	TiO_2_ nanoarrays with different morphologies in titanium.	TiO_2_ nanorods with a larger diameter promotes osteogenic differentiation of BMSCs and stimulates macrophage polarization to M2 generating an immune microenvironment.
Su et al., 2020 [69]	In vitro	Graphene oxide (GO) coating in titanium surfaces	Manipulate the polarization of macrophages and the expression of inflammatory cytokines. lmmunomodulatory effects in osteogenesis.
Li et al., 2020 [70]	In vitro	Thermo-sensitive hydrogel on anodized Ti surfaces	Macrophages polarize toward the M2 phenotype, promotes tissue repair and osteoblast differentiation.
Chen et al., 2022 [71]	In vitro	Curcumin loaded through polydopamine (PDA) onto copper-bearing titanium alloy (Cu-Ti)	Immune regulation of macrophages through regulation of their polar differentiation.
Liu et al., 2024 [72]	In vitro and in vivo	Metal phenolic nanocoating consisting of tannic acid and strontium on Ti substrates	Antioxidant properties, accelerated osteogenic differentiation, inhibition of inflammatory responses.
Doadrio et al., 2015 [73]	In vitro	TNTs and ibuprofen	Confirmation of the ability of TNTs to act as an intelligent nanomaterial
Shen et al., 2020 [74]	In vitro	TNT-Cht and DEX	Enhanced proliferation and differentiation of osteoblasts, suppressed production of nitric oxide (NO) and pro-inflammatory cytokines from macrophages.
Luo et al., 2019 [75]	In vitro	MSNs + DEX	M2-polarization of macrophages, favorable osteogenesis but dose dependent toxicity.
Wei et al., 2020 [76]	In vitro and in vivo	PLGA nanofibers loaded with aspirin	In vitro inhibition of M1 polarization and increased proliferation and differentiation of MSCs to osteoblasts, in vivo enhanced osseointegration.
You et al., 2022 [77]	In vitro and in vivo	PLGA loaded with aspirin in 3D printed Ti alloy implants	In vitro enhanced M2 gene and protein expression and in vivo superior osseointegration.
Zhao et al., 2021 [78]	In vitro	Double layer customization on TNTs Internal layer: MSC-derived exosomes on polydopamine External layer: 3-day differentiated MSC-derived exosomes on hydrogel	Enhances the migration and osteogenic differentiation of hBMSCs. Modulation of macrophage polarization.
Jayasree et al., 2023 [79]	In vitro	TNTs loaded with microvessels (MVs)	Controlled local release pattern for up to 7 days. Reduction in the production of pro-inflammatory cytokines in keratinocytes.

**Table 3:** Studies with nanomaterials for the prevention of peri-implantitis. Abbreviations: Ti: Titanium; AgNPs: Silver Nanoparticles; TNTs: Titania Nanotubes; AMP: Antimicrobial peptides; PLGA: Polylactic-co-glycolic acid; Zn: Zinc; ZnO: Zinc Oxide; Ag: Silver; Cu: Copper; Bis: Bismuth; HA: Hydroxyapatite; Cht: Chitosan; PVA: Polyvinyl Alcohol; MSNs: Mesoporous Silica Nanoparticles; OCT: Octenidine; PDA: Polydopamine.

Studies with Nanomaterials for the Prevention of Peri-Implantitis			
Study	Method	Nanomaterial	Result
Puckett et al., 2010 [89]	In vitro	Nanorough Ti surfaces from electron beam evaporation	Decreased bacterial adhesion especially of *S. aureus*, *S. epidermidis* and *P. aeruginosa*.
Cao et al., 2011 [90]	In vitro	AgNPs	Inhibition of *S. aureus* and E. coli growth and enhanced antibacterial activity of the surface due to micro galvanic effects. The amounts of *S. aureus* and *E. coli* on 0.5h-Ag-PIII are reduced by approximately 93% and 95% after 24 h.
Zhu et al., 2015 [91]	In vitro	AgNPs	Anti-bacterial activity against gram-positive *S. aureus* and gram-negative *F. nucleatum*. The antibacterial activity of Ag NPs against *F. nucleatum* was superior to *S. aureus*.
Lampé et al., 2019 [92]	In vitro	AgNPs	64.6% of antibacterial effect was noted for the nanoparticle-covered samples.
Liu et al., 2017 [93]	In vitro	AgNPs contained in HA	Bacterial inhibition for percentage of 2% silver.
Gosau et al., 2015 [94]	In vitro	Nanocrystalline Ag, Cu and Bis coating	Favorable anti-bacterial effects, but cytotoxicity for Cu.
Hameed et al., 2018 [95]	In vitro	CuNPs	Enhanced antibacterial effect against *P. gingivalis*.
Liu et al., 2015 [96]	In vitro	Polydopamine (PDA) coated zirconia	Increased cell adhesion and proliferation. The number of adherent bacteria decreased significantly on zirconia after PDA coating. The PDA coated zirconia showed both lower percentages of S. gordonii (0.91 ± 0.16%) and *S. mutans* (1.85 ± 0.48%) than the pristine zirconia (1.73 ± 0.32% and 3.06 ± 0.47%) (*p* < 0.01).
Zhao et al., 2011 [97]	In vitro	TNTs loaded with AgNPs	TNTs kill planktonic bacteria for the first days after surgery and inhibit bacterial adhesion for 30 days.
Huo et al., 2013 [98]	In vitro	TNTs loaded with Zn	Good intrinsic antibacterial properties with simultaneous favorable soft and hard tissue integration.
Wang et al., 2020 [99]	In vitro and in vivo	graphdiyne (GDY) composite TiO_2_ nanofiber coating	Increased photocatalysis and prolonged antibacterial ability, especially against methicillin-resistant staphylococcus aureus (MRSA). ROS release from this system prevented the formation of biofilm. In standard plate counting assay tests, the number of colonies of the TiO_2_/GDY + UV group reduced by 98% compared to that of the group not treated with UV
Gulati et al., 2012 [100]	In vitro	TNTs loaded with indomethacin and covered by chitosan/PLGA	Extended drug release properties, favorable bone cell adhesion and improved anti-bacterial properties.
Kumeria et al., 2015 [101]	In vitro	TNTs decorated with micelles loaded with gentamicin and covered by chitosan/PLGA	Long term and improved anti-bacterial properties, prevention of biofilm formation.
Baghdan et al., 2022 [102]	In vitro	PLGA loaded with norfloxacin on Ti discs	Up to 99.83% reduction in the number of viable bacterial colonies.
Ma et al., 2011 [103]	In vitro	TNTs loaded with AMPs	Reduction of gram-positive bacterium *S. aureus* levels and inhibition of bacterial adhesion on the implant surface. In survival assay tests, AMP loaded TNTs demonstrated bacterial killing with approximately 99.9% decrease. About 200-fold decrease of bacterial colonies was observed for the peptide-loaded groups compared with the groups without peptide.
Srivastava et al., 2024 [104]	In vitro	Macroporous Ti matrix is filled with mesoporous silica, coated with crosslinked chitosan releasing CHX	reduced numbers of bacterial growth compared to the uncoated Ti/SiO_2_ sample (*S. sobrinus*, *F. nucleatum*)
Cheng et al., 2019 [105]	In vitro	AgNPs on catechol-containing chitosan (CACS) coatings	Anti-bacterial properties of the system, both against gram-positive and gram-negative bacteria.
Mishra et al., 2017 [106]	In vitro	Cht-PVA-Silver nanocomposite coating	Better functional properties and enhanced bactericidal activity against *S. aureus* and *E. coli*.
Song et al., 2016 [107]	In vitro	Gelatin nanospheres loaded with antibiotics and encapsulated in chitosan matrix	Inhibition of bacterial growth. In inhibition zone tests the samples that contained moxifloxacin with or without gelatin nanospheres displayed an obvious inhibition zone whereas none of the groups with or without vancomycin induced the formation of an inhibition zone.
Choi et al., 2019 [108]	In vitro	AgNPs on PDA	Less bacteria colonization in Ag/PDA treated implants when compared with uncoated titanium surfaces, bacterial growth was found retarded in bacterial growth curves for *S. mutans* and *P. gingivalis*.
Palla-Rubio et al., 2019 [109]	In vitro	Silica—chitosan coating on Ti implants	Coatings with 5% and 10% of chitosan have particularly good bactericidal properties.
Xu et al., 2017 [110]	In vitro	MSNs loaded with OCT	Inhibition of bacterial adhesion was noted, especially for *S. mutans* and *E. coli*. The antibacterial ratios of *S. aureus* and *E. coli* were 21.5 ± 6.2% and 13.1 ± 4.8%, and 97.1 ± 0.8% and 86.3 ± 1.2%, in respect to MAO/Si substrates and MAO/Si/OCT substrates, respectively.
Li et al., 2017 [111]	In vitro	PSA nanoparticles, zinc oxide (ZnO) covered by a silica film on the outside and N-halamine polymer labeling	Excellent anti-bacterial activity against *P. aeruginosa*, *E. coli* and *S. aureus* with no obvious cytotoxicity.
Kulshrestha et al., 2014 [112]	In vitro	Graphene ZnO coating	Reduction in biofilm deposition.
De Leo et al., 2017 [113]	In vitro	Liposome coatings	The system can be utilized for the incorporation of various moieties with different polarities such as an antibiotics, anti-inflammatory drugs and protein like growth factors.

**Table 4:** Studies with nanomaterials for corrosion resistance. Abbreviations: Zr: Zirconium; TiNi: Titanium-Nickel; TNTs: Titania Nanotubes; Cu: Copper.

Studies with Nanomaterials for Corrosion Resistance			
Study	Method	Nanomaterial	Result
Indira et al., 2004 [116]	In vitro	ZrNPs loaded in TNTs	Enhanced corrosion resistance.
Al-Saady et al., 2023 [117]	In vitro	Titanium oxide nanotubes	Enhanced corrosion resistance.
Azari et al., 2023 [118]	In vitro	HA coating with intermediateTiO_2_ layer on Ti6Al4V substrates	Intermediate layer reduces the corrosion current by 65 percent and improves the corrosion resistance of monolayer HA-coated Ti-6Al-4 V alloy.
Shen et al., 2022 [119]	In vitro	Silicon nitride (Si_3_N_4_) nanoparticles	Corrosion tendency and corrosion rate of Si3N4-doped specimens were significantly reduced, with Si_3_N_4_ concentration dependence.
Afrouzian et al., 2021 [120]	In vitro	Silica coating (SiO_2_) on the surface of Ti6Al4V alloy via 3D printing	Promising tribological performance.
Hsu et al., 2021 [121]	In vitro	Silicon carbide (SiC) on titanium dioxide nanotubes (ATO)	Improved corrosion resistance.
Harb et al., 2020 [122]	In vitro	PMMA-TiO_2_ and PMMA-ZrO_2_ nanocomposite coatings with calcium phosphates in Ti_6_Al_4_V implants	Excellent corrosion resistance in SBF solution. PMMA-TiO_2_-βTCP coating presented low frequency impedance modulus of 430 GΩ cm^2^ unchanged for 21 days. (>100 GΩ cm^2^ in coatings indicate very good anticorrosion protection).
Kazemi et al., 2020 [123]	In vitro	Titanium Nitride (TiN)-HA multilayer composite in Ti_6_Al_4_V implants	Lowest corrosion current density and highest corrosion potential.
Aydin et al., 2021 [124]	In vitro	TiO_2_ nanotubes modifies with ZnO nanorods and AgNPs	ZnO-TiO_2_ nanotubes exhibited high resistance value at immersion of 7 days.
Xia et al., 2020 [125]	In vitro	C/Cu NPs	Improved mechanical properties and reduction of free copper ions. The Cu ion release was regulated by the galvanic corrosion effect of the system, with no additional cytotoxicity induced.
Zheng et al., 2008 [126]	In vitro	Zr coating in TiNi alloy implant	Reduced Ni ion release and improved corrosion resistance was noted for Zr coated substrates.
Yusuf et al., 2023 [127]	In vitro	Nano Mg-PSZ partially stabilized zirconia	The greater the concentration of magnesia (MgO) in doping the ZrO_2_, the greater the degradation resistance of Mg-PSZ in simulated body fluid (SBF) solution.
Zaher et al., 2024 [128]	In vitro	Amorphous calcium phosphate nanoparticles (ACP-NPs) in Ti bare	Increased corrosion resistance.

## Data Availability

No new data were created or analyzed in this study. Data sharing is not applicable to this article.
